# 421. Impact of SARS-CoV-2 Infection on Physical Fitness in Air Force Basic Trainees

**DOI:** 10.1093/ofid/ofad500.491

**Published:** 2023-11-27

**Authors:** Elena M Crouch, Joseph Marcus, Heather Yun, Theresa Casey, John Kieffer, Erin Winkler, Korey Kasper, John Kiley

**Affiliations:** San Antonio Uniformed Services Health Education Consortium, San Antonio, Texas; Brooke Army Medical Center, San Antonio, Texas; Brooke Army Medical Center, San Antonio, Texas; BAMC, San Antonio, Texas; BAMC, San Antonio, Texas; BAMC, San Antonio, Texas; 559th Trainee Health Squadron, San Antonio, Texas; BAMC, San Antonio, Texas

## Abstract

**Background:**

The impact of SARS-CoV-2 infection on physical fitness in previously healthy adults is not well known. In this study we assess the impact of SARS-CoV-2 infection on the physical fitness test scores of Air Force basic trainees

**Methods:**

SARS-CoV-2 testing data and trainee fitness test scores for the calendar year 2021 were obtained from US Air Force basic military trainees. Trainees perform a standardized fitness test involving push-ups, sit-ups, and a 1.5 mile run at the beginning and end of their training. Basic trainees who performed two fitness tests in 2021 and also tested positive for SARS-CoV-2 were defined as the infected (Inf) cohort and were 1:1 matched by sex to an uninfected control (Ctl) group of basic trainees. Categorical variables were compared using Chi-squared or Fisher’s exact test and continuous variables were compared using Mann-Whitney-U.

**Results:**

23,450 basic trainees performed a fitness test in calendar year 2021, 975 (4%) of whom tested positive for SARS-CoV-2. 621 (64%) had completed two fitness tests during the defined study period and were included in the Inf cohort. There were 96 females (15.5%) in each group and 525 males (84.5%) in each group. There was no difference in body mass index between the Inf and the Ctl group (24.0 (IQR 21.8 – 36.0) vs 24.3 (IQR 21.7-36.9), p=0.253). The Inf group had a higher rate of failing their physical fitness test at the end of training when compared to Ctl (15.7% vs. 4.3%, p< 0.001). When comparing those who passed their first fitness test and went on to fail their second fitness test, this occurred more frequently in the Inf group (2.3% vs. 0.81%, P = 0.037). When comparing trainees who failed their first fitness test and went on to pass their second fitness test, this occurred more frequently in the Ctl group (46.3% vs. 39.6%, P = 0.016). Amongst the Inf group, there were no differences in 2^nd^ test failure rates when comparing symptomatic to asymptomatic trainees (16.9% vs. 11.8%, P = 0.143).

**Conclusion:**

SARS-CoV-2 infection was associated with an increased risk of physical fitness test failure as well as conversion from a passing to failing test score. The presence of symptoms was not associated with failing test two. Possible confounders include lost fitness training time due to illness.

**Disclosures:**

**All Authors**: No reported disclosures

